# Synthesis of Copper Nanoparticles in Ethylene Glycol by Chemical Reduction with Vanadium (+2) Salts

**DOI:** 10.3390/ma9100809

**Published:** 2016-09-29

**Authors:** Andrea Pietro Reverberi, Marco Salerno, Simone Lauciello, Bruno Fabiano

**Affiliations:** 1 DCCI—Department of Chemistry and Industrial Chemistry, via Dodecaneso 31, Genova 16145, Italy; 2 Nanophysics Department, Istituto Italiano di Tecnologia, via Morego 30, Genova 16163, Italy; marco.salerno@iit.it; 3 Nanochemistry Department, Istituto Italiano di Tecnologia, via Morego 30, Genova 16163, Italy; simone.lauciello@iit.it; 4 DICCA—Department of Civil, Chemical and Environmental Engineering, Chemical Engineering Section, via Opera Pia 15, Genova 16145, Italy; brown@unige.it

**Keywords:** copper, nanoparticles, bottom-up, ethylene glycol, reduction, surfactant

## Abstract

Copper nanoparticles have been synthesized in ethylene glycol (EG) using copper sulphate as a precursor and vanadium sulfate as an atypical reductant being active at room temperature. We have described a technique for a relatively simple preparation of such a reagent, which has been electrolytically produced without using standard procedures requiring an inert atmosphere and a mercury cathode. Several stabilizing agents have been tested and cationic capping agents have been discarded owing to the formation of complex compounds with copper ions leading to insoluble phases contaminating the metallic nanoparticles. The elemental copper nanoparticles, stabilized with polyvinylpyrrolidone (PVP) and sodium dodecyl sulphate (SDS), have been characterized for composition by energy dispersive X-ray spectroscopy (EDS), and for size by dynamic light scattering (DLS), and transmission electron microscopy (TEM), giving a size distribution in the range of 40–50 nm for both stabilizing agents. From a methodological point of view, the process described here may represent an alternative to other wet-chemical techniques for metal nanoparticle synthesis in non-aqueous media based on conventional organic or inorganic reductants.

## 1. Introduction

Inorganic nanoparticles found several applications in physics, medical sciences and chemical engineering for their optical, therapeutical [1] and catalytic applications [2]. In particular, the manufacture of composite materials [3] has been revamped by nanotechnology owing to their promising applications to sensors and membrane separation processes [4]. Recently, nanotechnologies for hydrogen production [5] led to surprising results in photocatalytic conversion of pollutants as hydrogen sulphide [6].

Except for biotechnological synthesis methods, generally representing a recent and expanding world apart from traditional manufacturing techniques, we observe that physical and chemical methods for nanoparticle synthesis are essentially based on top–down and bottom–up techniques [7], according to a well-known classification currently accepted in the scientific community [8]. Top–down techniques in wet-chemical synthesis, despite an intrinsic simplicity in their processing steps, did not find extensive application owing to some difficulties in controlling shape and size distribution function of the nanostructured phase. In fact, size polydispersity represents a crucial drawback in many fields, as in optics and electronics, where usually both mean and standard deviation of diameters have to be minimized. On the contrary, bottom–up methods have been addressed by many researchers who worked out a large number of different strategies for metal nanoparticle synthesis by chemical routes, as these represent an economically viable path for manufacturing technology transfer on an industrial scale [9], posing less demand as well in terms of workplace and process nanosafety [10]. 

Copper nanoparticles (Cu-NPs), together with silver and gold, have long been objects of intense investigations for their peculiar surface plasmon resonance (SPR) occurring in the range of visible light [11]. Likewise, other noble and non-noble metals, such as Pt, Pd and Bi, showed analogous properties despite receiving less attention [12]. SPR has been exploited in the manufacturing of highly selective colorimetric sensors for amino-acid detection [13], in the fabrication of optical filters [14] and in humidity measurements [15]. Moreover, Cu-NPs proved to be highly efficient in many catalytic processes concerning the production of fine and base chemicals [16]. For a comprehensive review on this field, we refer the reader to the paper of Gawande et al. [17] and the references cited therein. 

In the specific context of Cu-NP synthesis by wet-chemical methods [18], the variants among processes differ from one another according to the use of the solvent embedding the precursor, the type of reductant donating electrons to the precursor’s cations, possibly utilizing environmental friendly agents [19], the kind of surfactants or capping agents adopted to prevent the particles from aggregation, and the choice of operative conditions aiming at maximizing the reaction yield in a short time.

In many cases, organic solvents like polyols [20] have been preferred to water, as their viscosity proved to be effective in minimizing particles aggregation and in preventing them from oxidation, which is one of the more crucial drawbacks affecting Cu-NP wet-chemical methods.

Sodium borohydride (NaBH_4_), hydrazine (N_2_H_4_), sodium hypophosphite (NaH_2_PO_2_) and several organic compounds including ascorbic acid, diethanolamine and isopropanol [21] are the mainly adopted reductants in Cu-NP chemical synthesis, with a variable choice of temperature values according to the chemical kinetics typical of the specific reaction. As a rule of thumb, fast kinetics are generally preferable when small (≤50 nm diameter) nanoparticles are needed, as short nucleation times promote a simultaneous particle capping by surfactants damping a competitive aggregation. To this purpose, the higher strength of reductants is often associated with faster kinetics, spanning in a range of seconds in case of NaBH_4_ up to some hours in the case of ascorbic acid or other organic electron donors. 

The choice of surfactants is wide, including ionic and non-ionic chemical species having a basic role in stabilizing the Cu-NPs for even months [22]. Cationic surfactants such as quaternary ammonium halides containing a non-polar aliphatic chain, and anionic surfactants such as alkali metal alkyl sulfonates, showed excellent properties in aqueous and organic media. Sometimes, the latter have been used for their joint properties of solvents and surfactants. As a general trend, the Cu-NP diameter increases for growing precursor concentrations and decreases at higher surfactant concentrations. 

In this paper, we propose a process for the synthesis of Cu-NPs, which is traditional in the choice of solvents and stabilizing agents but is atypical in the choice of reductant. In fact, to the best of our knowledge, this is the first case where divalent vanadium salts have been used for Cu-NP preparation in an organic solvent. The choice of this reductant is motivated by several reasons, namely:

- vanadium (+2) sulphate, different from other routinely adopted inorganic reductants with basic properties, has an acid behavior, and this proved to be beneficial in hindering the formation of a surface layer of mixed oxides on Cu-NPs [23];
- vanadium (+2) sulphate reduces copper salts at room temperature with fast kinetics comparable to those typical of NaBH_4_, thus allowing much lower temperatures with respect to those typical of processes where conventional reductants operating in non-aqueous solvents are used;
- vanadium (+2) cations interfere with copper cations in the process only in some cases, namely when non-ionic stabilizing agents are used to prevent nanoparticles aggregation in a non-aqueous solvent embedding the precursor. This side effect will be discussed in the following section.

The paper is organized as follows. In Section 2, the results are presented and the nanosized phase is characterized by several techniques aiming at determining the surface geometry and the chemical composition of the nanoparticles surface. In Section 3, the experimental setup is described, giving some details concerning the preparation of the reductant requiring some technical tricks of electrochemistry. Furthermore, the specific chemistry of reduction is discussed, the apparatus for the synthesis of reductant is described and the choices of the relevant stabilizing agents are motivated. Finally, the conclusions are drawn and possible applications for future works are proposed. 

## 2. Results and Discussion

**Figure 1:**
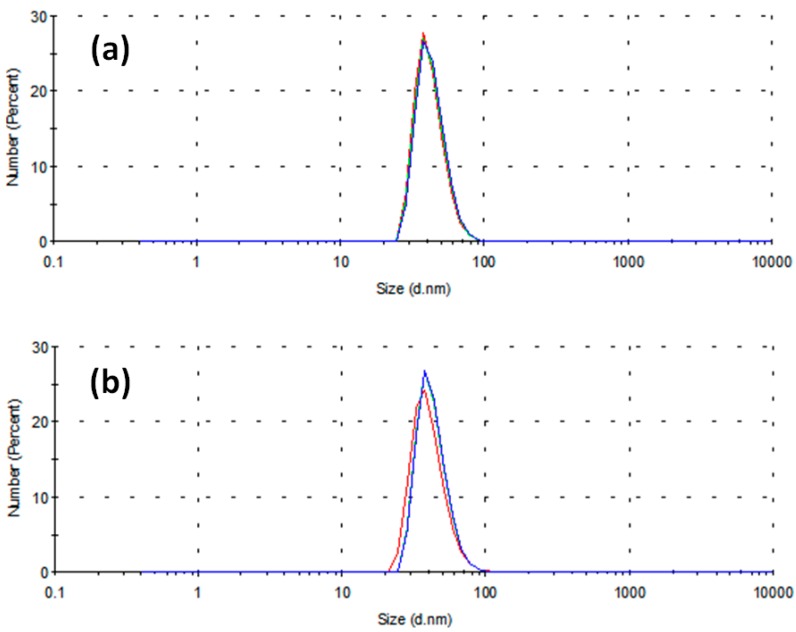
Distribution of particle diameters populations by number versus diameters obtained by DLS. (**a**) Specimen obtained using PVP 40 k as stabilizing agent; average diameter = 41.8 nm; and (**b**) specimen obtained using SDS as stabilizing agent; average diameter = 40.5 nm. In each plot, the curves refer to three different sampling times differing by 5 min from one another.

**Figure 2:**
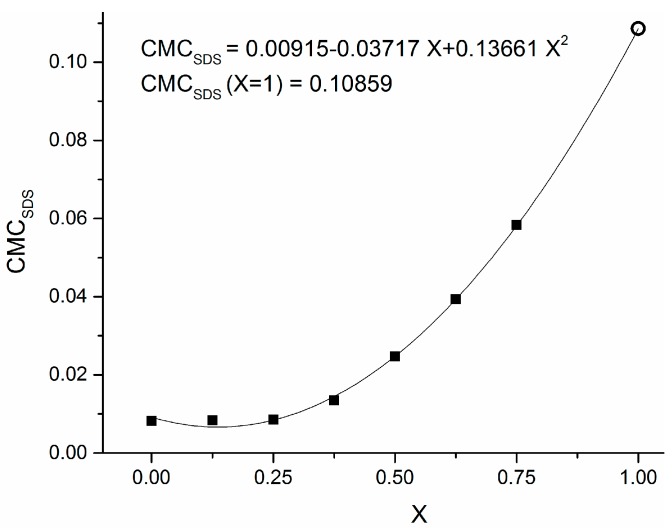
Plot of critical micellar concentration of SDS in a mixture of EG–H_2_O versus the volume fraction x of EG. The solid squares are data taken from Ref. [29], while the hollow circle is an extrapolated value obtained using the parabolic fitting curve for x = 1.

Figure 1 reports the distribution of particle diameters population by number in the case of two different stabilizing agents, namely 0.1 g PVP (Figure 1a) and 0.005 M SDS (Figure 1b) in 10 mL of 0.001 M CuSO_4_ precursor in ethylene glycol (EG). The three different curves reported in each panel refer to three sequential time determinations by DLS, differing by 5 min from one another. It is worth remembering that the capping activity of SDS operates according to two different mechanisms, depending on whether its concentration C_SDS_ is smaller or greater than a threshold value CMC_SDS_, defined as critical micellar concentration (CMC) [24]. It has been reported that, when C_SDS_ < CMC_SDS_, the surfactant molecules tend to direct their polar head toward the surface of a single nanoparticle. On the contrary, when C_SDS_ > CMC_SDS_, the surfactant molecules group into micelles whose polar surface acts as a growing site for metal nanoparticles, forming a hollow agglomerate surrounding the aforementioned micelle [25]. Ali et al. [26] considered aqueous solutions of SDS in the presence of amino acids and pointed out that the CMC value for SDS depends on many factors, such as the temperature and other properties related to the chemical structure of solvent, surfactant and other dissolved compounds. It is intriguing to observe that almost all previous works on critical phenomena in micellar transitions of surfactants are focused on aqueous dispersions [27], but there are rare exceptions. In fact, Yuan et al. [28] observed that the CMC of SDS in polyethylene glycol-water mixtures is far below its critical value in aqueous solutions. On the contrary, old studies in water–EG mixtures up to 75% of EG [29] showed a high increase in CMC_SDS_ for concentrated EG solutions in water. Therefore, in the absence of reference data concerning CMC for SDS dissolved in pure EG, we used his findings to extrapolate the CMC_SDS,EG_ value for pure EG, according to the scatter graph reported in Figure 2, obtaining a value of CMC_SDS,EG_ ≅ 0.11.

The above unexpected value is more than one order of magnitude greater than the well-known critical value CMC_SDS,W_ ≅ 8 × 10^−3^ M corresponding to water solutions [30]. This result can explain why, when we repeated the Cu-NP synthesis with 0.05 M SDS by DLS characterization, we received no significant difference in the particle population curves with respect to the sample with 0.005 M SDS: in both cases, we are largely below the estimated value of CMC_SDS,EG_.

**Figure 3:**
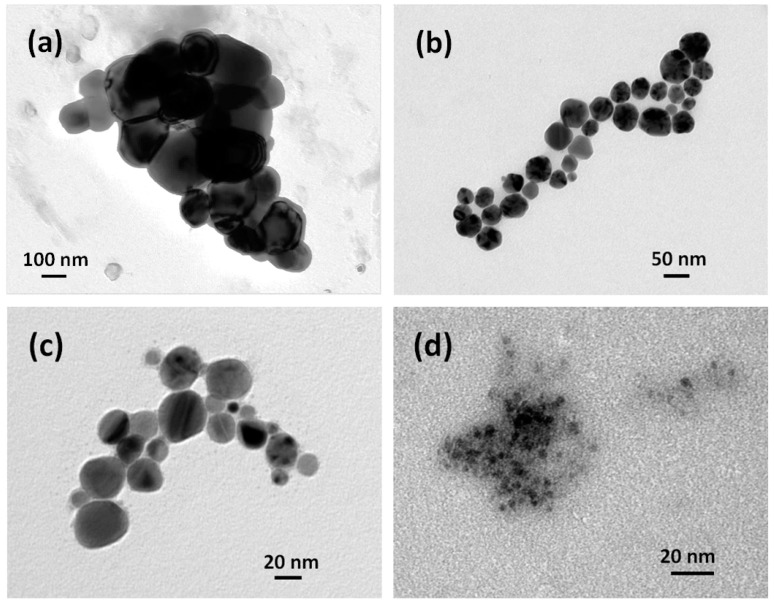
TEM images obtained according to different operating conditions, using (**a**) no surfactant; (**b**) PVP and stoichiometric reductant; (**c**) SDS and stoichiometric reductant; and (**d**) PVP and one-third of the stoichiometric amount of reductant.

In Figure 3a, a TEM image is shown of the nanoparticles synthesized without surfactant. The typical nanoparticle size is in the range of 100–300 nm. In Figure 3b, an image representative of the same experimental situation as described in Figure 1a, namely in the case of PVP as a stabilizing agent, appears. The typical size is smaller here, approximately 50 nm, consistent with the DLS data in Figure 1. A halo surrounding the nanoparticles appears, which is an effect of the residual presence of stabilizing agent adsorbed on copper surface, whose removal proved to be difficult even after washing with ethanol followed by drying under vacuum. The nanoparticles are approximately spherical in shape, with the rare occurrence of sharp corners probably due to facets of polyhedra. A small number of particles (approximately below 3%) exhibit perfect cubic shape (data not shown). Figure 3c reports results not much different from those in Figure 3b, though it refers to SDS as in the experimental data of Figure 1b. Again, the sizes are consistent with the curves reported in DLS analysis, with a slight decrease in polyhedral elements and a greater number of round-shaped single particles. 

Up to now, we have presented the results keeping the concentration of the reductant (VSO_4_) at its stoichiometric value. In Figure 3d, the VSO_4_ concentration has instead been kept at one-third of the stoichiometric value required by 10 mL of 0.001 M Cu^2+^ precursor with 0.1 g PVP. A marked reduction in average diameter of the nanoparticles can be seen by simple inspection, in contrast with the well-known kinetic theory where small particle diameters are associated to high reductant concentration, promoting instantaneous nucleation while contrasting growth. A possible interpretation of this phenomenon can be given taking into account some aspects of this process that can be summarized as follows:

- The capping activity of a non-ionic stabilizing agent toward Cu^2+^ ions is due to the presence of active sites on its molecule as electron-pair donors to the Cu^2+^ ion itself. The oxygen atom in the ketonic group of PVP, with its double pair of free electrons, is one of the best candidates for this role.
- Vanadium, being a transition element as copper, may compete against copper in forming coordination bonds with the aforementioned electron-pair donors, possibly deactivating the PVP in its capping activity. Therefore, this latter effect might be damped at lower concentrations of reductant, and this fact might explain how smaller diameters have been obtained in this condition. To the best of our knowledge, this is the first case when a reductant containing a transition element is adopted in the synthesis of metal nanoparticles.

**Figure 4:**
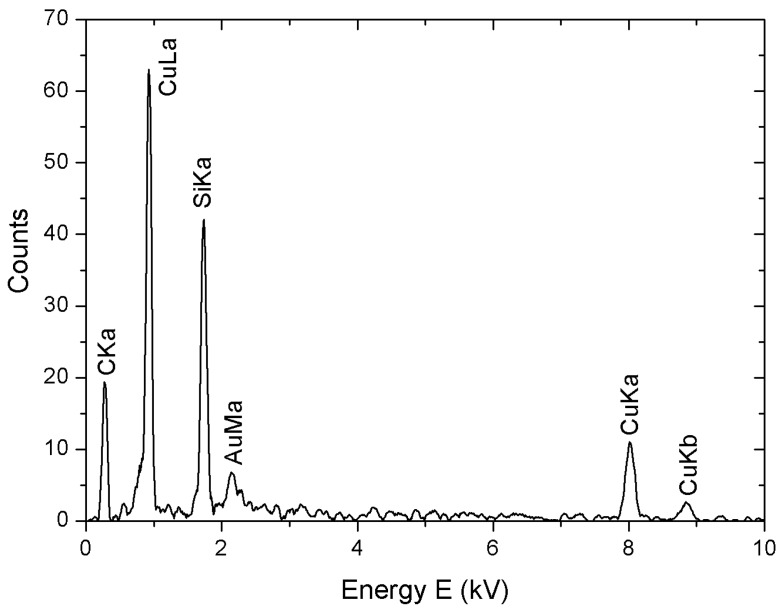
EDS analysis of a solid phase obtained by reduction without using stabilizing agents.

In Figure 4, we report the results of an EDS obtained by scanning electron microscopy (SEM) analysis of Cu-NPs carried out in the absence of stabilizing agents. The peaks are related to the chemical composition of both nanoparticles and substrate, made of Si, C and Au. No oxygen was detected, excluding the presence of copper oxides like CuO or Cu_2_O.

## 3. Materials and Methods

### 3.1. Reduction Process

**Figure 5:**
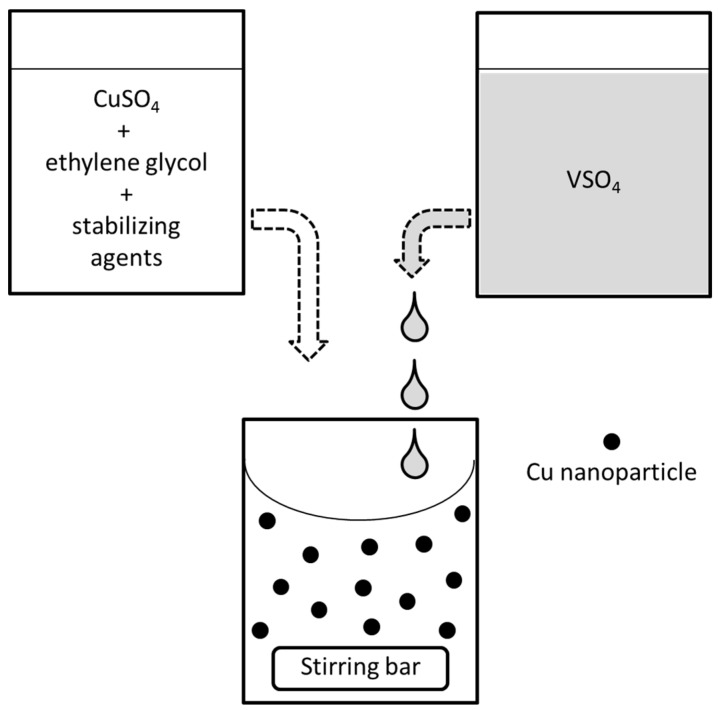
Scheme of the process here adopted for the synthesis of Cu-NPs.

The synthesis of zerovalent Cu-NPs relies upon a reduction process having copper sulphate (CuSO_4_·5H_2_O, >99%, Sigma-Aldrich, Milano, Italy) as precursor, which is dissolved in EG (C_2_H_6_O_2_, 99.8%, Sigma Aldrich, Milano, Italy). For all samples, the concentration of the precursor is kept fixed at 1 × 10^−3^ M. The choice of suitable stabilizing agents is an important constraint, as cationic stabilizing agents like quaternary ammonium salts often give white precipitates with Cu cations in wet-chemical Cu-NP synthesis based on reduction processes. This phenomenon has been previously reported by De and Mandal [31], who observed the formation of a white crystalline phase when cetyl-trimethylammonium bromide (CTAB) is used as capping agent in the presence of Cu ions. The composition of this precipitate is still partially obscure, but it seems to be due to formation of an organic Cu-CTAB complex, soluble in organic solvents like acetonitrile or chloroform and insoluble in solvents with greater polarity. In the present experiments, the same undesired side-reaction leading to formation of an analogous crystalline solid phase was observed when using cationic stabilizing agents with long hydrophobic chain, namely CTAB and myristyl-trimethylammonium bromide (MTAB), or even with short hydrophobic chain like tetrabutylammonium bromide (TBAB). For this reason, quaternary ammonium capping agents have been avoided; in their place, PVP 40 k with 40 kDa molecular weight ((C_6_H_9_NO)_n_, 95%, Sigma-Aldrich, Milano, Italy) or SDS (CH_3_(CH_2_)_11_OSO_3_Na, 99%, Sigma-Aldrich, Milano, Italy) are chosen, and mixed with the precursor in EG giving the solution described by letter A in Figure 5. 

A separate solution B of vanadium (II) sulphate VSO_4_ in water is prepared according to methods that will be described in the following section. In particular, solution B is added dropwise to 10 mL of solution A in a well-stirred vessel at room temperature. The stoichiometric amount of reductant corresponds approximately to 0.3 mL of VSO_4_ solution. The color of the mixed solutions changes immediately, shifting from a colorless to a deep purple-red dispersion, which is transparent by transmission and brick-red opaque by visible light reflection, following a characteristic trend highly suggestive of zerovalent Cu^(0)^ formation according to the reaction:

CuSO_4_ + 2VSO_4_ → Cu^(0)^ + V_2_(SO_4_)_3_. (1)

### 3.2. Preparation of the Reductant

Vanadium divalent compounds, like VSO_4_, have a high tendency to combine with oxygen, both atmospheric or dissolved in solvents, giving V^+3^ and finally vanadyl (V^+4^) compounds. Therefore, almost all methods reported in literature for their synthesis from vanadium compounds at higher oxidation states are often tricky [32], as they require two precautions that, up to now, have been considered as indispensable, namely:

- in all situations, an inert atmosphere preserving from oxidation is generally needed;
- if an electrochemical process is adopted in aqueous solvent, a cathode having the highest overvoltage to the hydrogen discharge has to be adopted in order to maximize the current yield toward the vanadium reduction. As a consequence, a mercury, an amalgamated-metal cathode or a surface-pretreated lead cathode represent a basic choice to fulfil this stringent constraint [33].

In the present study, two different processes for the synthesis of VSO_4_ were used. In the former, vanadium pentoxide (V_2_O_5_, >99.6%, Sigma-Aldrich, Milano, Italy) finely dispersed in water is reduced by zinc granules (Zn, >99.8%, Sigma-Aldrich, Milano, Italy) in the presence of sulphuric acid (H_2_SO_4_, 98%, Sigma-Aldrich, Milano, Italy) as follows [34]:

V_2_O_5_ + 3Zn + 5H_2_SO_4_ → 2VSO_4_ + 3ZnSO_4_ + 5H_2_O (2)

The previous reaction is the result of several intermediate steps, where V^+5^ is converted first into vanadyl ions, then into V^+3^ ions, and finally reaches the dipositive state in the form of VSO_4_. Each oxidation state of vanadium cation is characterized by a typical color that makes it possible to monitor the attainment of the final V^+2^ configuration, giving a stable violet solution. This method suffers from two main drawbacks, namely:

- V_2_O_5_ in crystalline form is only sparingly soluble in water and a considerable amount of zinc is attacked by H_2_SO_4_, giving off hydrogen instead of taking part in Reaction (2).
- The presence of ZnSO_4_, which is dissolved together with VSO_4_, may trigger the formation of insoluble double zinc–vanadium sulphates. Regretfully, this insoluble phase may contaminate the Cu-NPs produced by Reaction (1).

**Figure 6:**
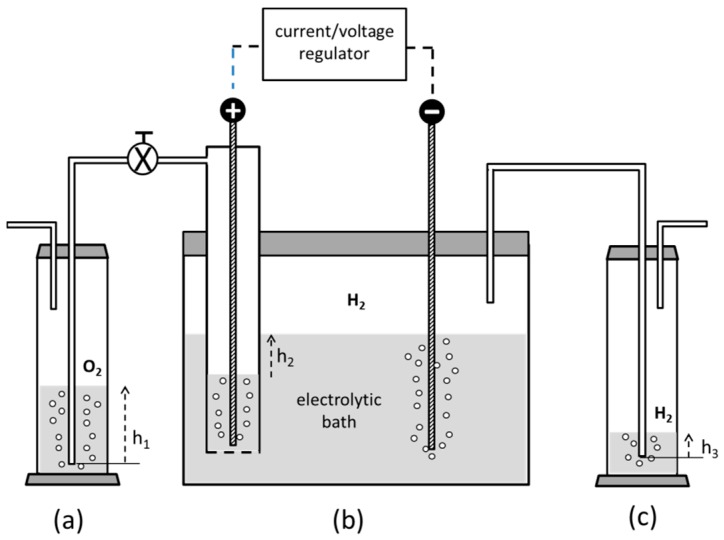
Scheme of the experimental apparatus for the preparation of the reductant (VSO_4_). (**a**,**c**) are Drechsel bottles where the gases produced at the anodic and cathodic compartments of the electrolytic cell; (**b**) are made to bubble in water. The difference in liquid height h_2_ between the interior and the exterior of the anodic compartment is regulated by properly setting the levels h_1_ and h_3_ at the beginning of the process.

For these reasons, this scheme was dropped and a more reliable process, carried out in an electrochemical cell described in Figure 6, was ultimately adopted with simplified technical tricks and satisfactory results [35].

The electrolyte is initially made of V_2_O_5_ in water containing a 10% excess of H_2_SO_4_ with respect to its stoichiometric amount, as in the former scheme. A lead anode is set in a compartment sealed at its bottom by a fritted glass, allowing for ionic exchange but avoiding mixing of the anolyte solution, rich of dissolved oxygen, with the outer electrolytic bath. Oxygen and hydrogen, produced at the anode and cathode, respectively, are conveyed externally to two Drechsel bottles and made to bubble in water. The difference in water level heights between the two bottles allows for controlling the liquid hold-up contained in the anode compartment, where the sulphate anions tend to concentrate, thus preventing the anolyte from flooding at higher currents. During the electrolysis, hydrogen increasingly occupies the volume above the electrolyte level, spontaneously creating an inert atmosphere with no need of venting inert gases inside the cell, unlike most works in literature. Moreover, despite many techniques being based on the use of mercury or anodized lead superficially coated by PbO_2_ at the cathode, we simply adopted pure lead for both electrodes without any surface pre-conditioning. This design strategy is in line with the inherent safety approach according to the guideword “substitution”, aiming at the use of plant/process safer materials [36]. The cathodic reactions carry out a sequential reduction of the pentavalent vanadium according to the following steps:

V_2_O_5_ + 2H_2_SO_4_ +2e^−^ → 2VOSO_4_ + 2OH^−^ + H_2_O, (3)

2VOSO_4_ + H_2_SO_4_ + 2e^−^ → V_2_(SO_4_)_3_ + 2OH^−^, (4)

V_2_(SO_4_)_3_ + 2H_2_O + 2e^−^ → 2VSO_4_ + H_2_SO_4_ + 2OH^−^. (5)

During Reaction (3), the electrolytic bath is kept under vigorous agitation by magnetic stirring until the solution becomes blue and transparent. Reactions (3) and (4), which are acid-consuming, do not require a careful current and temperature control, whose values may reach 0.5 A/cm^2^ and 60 °C, respectively. However, when the electrolyte assumes a dark-green color at the beginning of Reaction (5), small current densities not exceeding 0.05 A/cm^2^ and T < 30 °C have to be kept to maximize step (5) with respect to the competitive hydrogen production. Moreover, low cathodic current densities are beneficial, since an unwanted electrolyte temperature rise, typical of higher current densities, may likewise decrease the hydrogen overvoltage at the cathode, lowering the yield of Reaction (5). As a precaution, an external water cooling equipment is recommended at this last reduction stage [33]. For an initial concentration of 2 g V_2_O_5_ in 300 cm^3^ of electrolyte, the process is completed after approximately 48 h, when the electrolyte color remains permanently violet, which is typical of VSO_4_.

The presence of VSO_4_ can be ascertained by standard titration using a ferric salt solution in the presence of neutral red as a visual indicator, according to the method of Mittal and Mehrotra [37]. The solution of VSO_4_, when extracted from the electrolytic bath, must be used at once in Reaction (1) or stored in a reservoir out of air contact.

In order to ensure a satisfactory reproducibility of experimental data, we stress that the VSO_4_ solution should be employed after its color is asymptotically stable in time. Therefore, a good rule would be using this reagent after one day of buffer current is passed in the cell, even though no color change occurs to be sure that the reduction of vanadium to its divalent state has been completed.

### 3.3. Characterization Techniques

For the nanoparticle size, both dynamic light scattering (DLS) and transmission electron microscopy (TEM) were used. DLS was carried out by means of a Zetasizer Nano ZS (Malvern Instruments, Malvern, UK). The just synthesized colloidal solutions were typically diluted by a factor of three and measured in disposable polymethyl methacrylate (PMMA) cuvettes. The TEM was carried out on a JEM 1011 (Jeol, Tokyo, Japan) at an accelerating voltage of 100 keV, on samples of colloidal Cu-NP solution drop-cast on copper grids with 300 mesh. The EDS analysis of SEM was carried out on a JSM-6490LA (Jeol, Tokyo, Japan) working at 20 keV, on samples of colloidal Cu-NP solution drop-cast on pieces of Si wafer and sputter-coated with 3 nm Au, with aperture 2 and at a working distance of 10 mm.

## 4. Conclusions

A method for the synthesis of copper nanoparticles dispersed in ethylene glycol has been proposed, where a new type of reductant proved to have fast reaction kinetics at room temperature in the presence of two different stabilizing agents. From a methodological point of view, we can draw the following conclusions:

The preparation of this reagent by electrochemical methods has been realized in a simpler and inexpensive process as compared to those adopted in the existing literature. In fact, neither elemental mercury nor metals with specific pre-treatments have been adopted at the cathode, which is simply made of elemental lead. No inert gases have been used to ensure an inert atmosphere inside the cell, which was protected from external oxygen by a self-produced hydrogen as a side-reaction during the reduction of vanadium cation. A suitable time trend of both current and temperature in the cell has been the sole control parameter for the synthesis of VSO_4_, which interferes in the preparation of Cu-NPs only with PVP, and in an easily controllable way.

The EDS/SEM analysis has proven that the elemental copper, which the nanoparticles are made of, is not significantly contaminated by its oxides CuO or Cu_2_O, which are often present in wet-chemical processes carried out in a basic environment and in the presence of air. This fact can be related to the oxide–scavenger properties of an acidic medium typical of a VSO_4_ solution.

The process proved to be suitable for the synthesis of copper nanoparticles at room temperature in a non-aqueous solvent, with similar results in terms of average diameters in the case of PVP and SDS as stabilizing agents. Moreover, mild operative conditions in terms of temperature can be adopted, with positive effects on limiting surface oxidation. This aspect is particularly important in a polyol synthesis method, where much higher temperatures are often required to carry out a Cu^2+^ ion reduction with traditional reagents.

## Abbreviations

The following abbreviations are used in this manuscript:

NPs	nanoparticles
DLS	Dynamic Light Scattering
SEM	Scanning Electron Microscopy
EDS	Energy Dispersive X-ray Spectroscopy
SPR	Surface Plasmon Resonance
TEM	Transmission Electron Microscopy
PVP	Polyvynilpyrrolidone
SDS	Sodium dodecyl sulphate
CTAB	cetyl-trimethylammonium bromide
MTAB	myristyl-trimethylammonium bromide
TBAB	tetrabutylammonium bromide
EG	ethylene glycol
CMC	critical micellar concentration
